# Impact of Sirtuin Enzymes on the Altered Metabolic Phenotype of Malignantly Transformed Cells

**DOI:** 10.3389/fonc.2020.00045

**Published:** 2020-02-14

**Authors:** Zsuzsanna Gaál, László Csernoch

**Affiliations:** ^1^Institute-Clinic of Pediatrics, Department of Physiology, University of Debrecen, Debrecen, Hungary; ^2^Department of Physiology, University of Debrecen, Debrecen, Hungary

**Keywords:** cancer, epigenetics, metabolism, sirtuin enzymes, personalized treatment

## Abstract

Sirtuins compose a unique collection of histone deacetylase enzymes that have a wide variety of enzymatic activities and regulate diverse cell functions such as cellular metabolism, longevity and energy homeostasis, mitochondrial function, and biogenesis. Impaired sirtuin functions or alterations of their expression levels may result in several pathological conditions and contribute to the altered metabolic phenotype of malignantly transformed cells in a significant manner. In the twenty-first century, principles of personalized anticancer treatment need to involve not only the evaluation of changes of the genetic material, but also the mapping of epigenetic and metabolic alterations, to both of which the contribution of sirtuin enzymes is fundamental. Since sirtuins are central players in the maintenance of cellular energy and metabolic homeostasis, they are key elements in the development of metabolic transformation of cancer cells referred to as the Warburg effect. Although its most well-known features are enhanced glycolysis and excessive lactate production, Warburg effect has several aspects involving both carbohydrate, lipid, and amino acid metabolism, among which different tumor types have different preferences. Therefore, energy supply of cancer cells can be impaired by a growing number of antimetabolite agents, for which appropriate vectors are strongly needed. However, data are controversial about their tumor suppressor or oncogenic properties, the biological effects of sirtuin enzymes strongly depend on the tissue microenvironment (TME) in which they are expressed. Immune cells are regarded as key players of TME. Sirtuins regulate the survival, activation, metabolism, and mitochondrial function of these cells, therefore, they are not only single elements, but key regulators of the network that determines anticancer immunity. Altered metabolism of tumor cells induces changes in the gene expression pattern of cells in TME, due to altered concentrations of metabolite cofactors of epigenetic modifiers including sirtuins. In summary, epigenetic and metabolic alterations in malignant diseases are influenced by sirtuins in a significant manner, and should be treated in a personalized approach. Since they often develop in early stages of cancer, broad examination of these alterations is required at time of the diagnosis in order to provide a personalized combination of distinct therapeutic agents.

## Introduction

First definition of epigenetics is derived from Conrad Hal Waddington (1942), who proposed it as “the causal interactions between genes and their products, which bring the phenotype into being” (1). Today, the term epigenetics involves all mechanisms that modify gene expression pattern without altering the sequence of the DNA (2). Key elements of epigenetic regulation are DNA methylation, histone modification, non-coding RNAs, and nucleosome remodeling. Epigenetic mechanisms are reversible and heritable (3). These features offer a great opportunity and at the same time, make high demands on personalized medicine.

First epigenetic alteration to be linked to cancer was global DNA hypomethylation described by Feinberg and Vogelstein in 1983 (3). To date, it has been confirmed that besides alterations in the DNA methylation pattern, changes in the histone code and in the expression levels of non-coding RNAs also contribute to the pathogenesis of malignant diseases in a significant manner.

**Figure 1:**
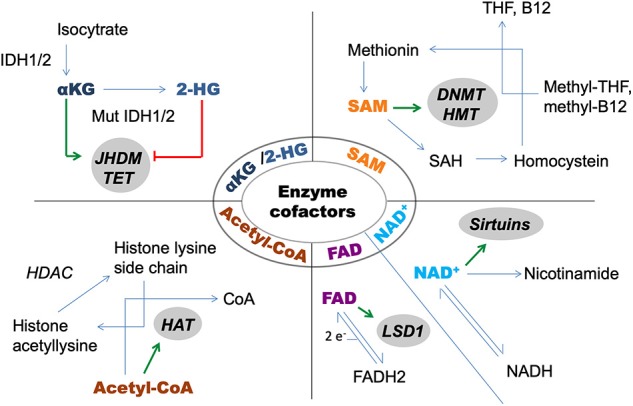
Metabolite cofactors of epigenetic modifier enzymes. 2-HG, 2-hydroxyglutarate; αKG, alpha-ketoglutarate; DNMT, DNA-methyltransferase; HAT, histone acetyltransferase; HDAC, histone deacetylase; HMT, histone methyltransferase; IDH, isocitrate-dehydrogenase; JHDM, Jumonji domain containing histone demethylase; LSD, lysinspecific demethylase; SAH, S-adenosyl homosysteine; SAM, S-adenosylmethionine; TET, DNA-hidroxymethylase enzyme; THF, tetrahydrofolate.

Among the several hallmarks that cancer cells acquire during tumorigenesis (4) altered metabolism is also a unique feature (5). The best-known characteristics of this special metabolic phenotype are increased rates of glycolysis and lactate production, which will be further detailed. Due to numerous interactions, epigenetic and metabolic alterations cannot be considered as independent players in the big puzzle of pathogenesis. The vast majority of enzymes that are responsible for catalyzing epigenetic alterations require metabolite cofactors [Figure 1; (6)]. Enzymes of the intermedier metabolism are also regulated by epigenetic alterations, for which an elegant example is the impact of histone acetyltransferase enzymes (HAT) on the activity of enzymes involved in glycolysis and fatty acid metabolism (7).

In recent years, numerous crosslinks have been established between epigenetics and metabolism in cancer, among which sirtuin enzymes have key significance.

## Sirtuins and Cancer

The currently known 18 histone deacetylase enzymes (HDACs) are divided into four groups, among which sirtuin enzymes, that are homologs to the yeast SIR2 protein (Silent Information Regulator 2), comprise group III, requiring NAD^+^ as a cofactor. They are a seven member family of protein deacylases and ADP-ribosyl-transferases with different targets, enzymatic activities, subcellular localizations, and regulatory mechanisms [Table 1; (8, 9)]. Since they are major hubs in the regulatory network of energy homeostasis and metabolism, sirtuins are potential therapeutic targets both in oncology and in the field of inborn errors of metabolism as well.

**Table 1:** Localization, enzymatic activity, and role of sirtuin enzymes in the regulation of metabolic pathways.

**Sirtuin**	**Primary localization**	**Enzymatic activities**	**Metabolism**
SIRT1	Nucleus	Deacetylation	Gluconeogenesis, glycolysis, insulin secretion, cholesterol, and fatty acid synthesis
SIRT2	Cytoplasm	Deacetylation Demyristoylation	Gluconeogenesis, triglycerid synthesis
SIRT3	Mitochondria	Deacetylation Decrotonylation	Glutamine metabolism, ketone body formation, Urea cycle, ß-oxidation of fatty acids
SIRT4	Mitochondria	Deacetylation ADP-ribosylation	Glutamine, leucine and carbohydrate metabolism, ß-oxidation of fatty acids
SIRT5	Mitochondria	Deacetylation Demalonylation Desuccinylation Deglutarylation	Glycolysis, TCA cycle, ketone body formation
SIRT6	Nucleus	Deacetylation Deacylation ADP-ribosylation	Glycolysis, gluconeogenesis, ß-oxidation of fatty acids
SIRT7	Nucleus	Deacetylation ADP-ribosylation	Lipid metabolism

In cancer, sirtuins have both oncogenic and tumor suppressor properties, however, data are controversial at several points. As an example for emphasizing the importance of tissue microenvironment, SIRT1 has been proposed both as a tumor suppressor and as an oncogene in different types of malignancies (8). SIRT4 acts as a tumor suppressor by regulating cell metabolism and inflammation as well (10). Oncogenic and tumor suppressor effects of sirtuins are also determined by the targets that they regulate. For example, SIRT7 was identified as a suppressor of MYC function, however, SIRT7 is essential in maintaining low levels of H3K18ac in cancer cells that is associated with poor clinical outcome (11, 12).

Altered expression levels of sirtuins contribute to chemoresistance and metastasis formation, and in some cases, they are associated with clinical outcome. In endometria carcinoma cell lines, SIRT1 overexpression enhanced resistance to paclitaxel and cisplatin (13). SIRT1 activation by MYC promotes resistance of FLT3-ITD-mutated acute myeloid leukemia stem cells to tyrosine kinase inhibitors (14). SIRT4 enzyme enhances the sensitivity of breast cancer cells to tamoxifen (15). SIRT4 also inhibits the migration and metastasis formation of thyroid cancer cells (16). SIRT6 promotes papillary thyroid cancer progression by inducing epithelial-mesenchymal transition (EMT) (17). In non-small cell lung cancer (NSCLC), expression of SIRT1 and SIRT2 is associated with poor prognosis (18).

Sirtuins are also involved in the pathogenesis of hematological malignancies. SIRT1 is overexpressed in human leukemia stem cells (LSC), and its inhibition suppressed proliferation of primitive progenitor cells and increased apoptosis in LSC (19). Due to our previous results, the expression level of SIRT6 enzyme negatively correlates with the level of the tumor suppressor miR-124 in acute myeloid leukemia (AML) (20).

The central role of sirtuin enzymes in the metabolism of cancer cells is confirmed by a growing number of evidences about their role in both promoting and inhibiting the Warburg effect (see below) in several tumor types. This strong impact on metabolism is highly associated with the numerous interactions of sirtuins with oncogenic and tumor suppressor proteins, microRNAs that regulate metabolism, and proteins involved in signal transduction pathways as well.

## The Warburg Effect

Nobel Laureate Otto Warburg observed in the 1920s that malignantly transformed cells prefer lactate production over oxidative phosphorylation regardless of the level of oxygen (21). The discovery of elevated glycolytic rate in tumor cells is still the basis of the worldwide used diagnostic method ^18^FDG PET (6).

Enhanced glycolysis is the most widely known feature of the altered metabolic phenotype of cancer cells. Glycolytic rate can be up to 200 times higher in malignantly transformed cells compared to healthy cells, to which up-regulation of GLUT transporters and overexpression of glycolytic enzymes also contribute (22, 23). MYC and HIF1α are both essential transcription factors in regulating the expression levels of enzymes involved in glycolysis, however, it is an important difference that while MYC enhances, HIF1α represses mitochondrial biogenesis (24, 25). Recently, many sirtuins have been proved to affect the activity of HIF1α: SIRT1 inhibits its transcriptional activity by deacetylation, SIRT2 increases its stability, while SIRT3 and SIRT7 destabilize it (26). The inhibition of HIF1α enzyme is a promising therapeutic target in several tumor types. Bortezomib, which was approved for the treatment of multiple myeloma in 2008, has also been proved to inhibit the transcriptional activity of HIF1α (27).

Though high rate of glycolysis results in excessive lactate production, systemic effects of lactate byproducts are rarely significant. However, type B lactate acidosis that develops under normoxic conditions is a rare, but life-threatening complication of hematological malignancies (28). Recent findings on a novel type of *in vivo* post-translational histone modifications, lysine lactylation, highlights the impact of elevated lactate production on gene expression (29). Histone lysine lactylation can directly promote the transcription of certain genes, however, its involvement in the metabolic switch of cancer cells has yet to be clarified (29).

Tumor suppressor proteins are also involved in the regulation of glycolysis. Excessive glycolysis and lactate production is counteracted by p53, which activates TIGAR enzyme and inhibits phosphoglycerate mutase 2 (PGM2). TIGAR is responsible for the cleavage of fructose-2,6-bisphosphate, the allosteric activator of phosphofructokinase I (PFK I) (23).

Opposite to oxidative phosphorylation that produces 36 molecules of ATP from one molecule glucose, glycolysis yields only 2. Despite this inefficient ATP generation, up-regulation of glycolysis provides cancer cells a number of advantages. Cancer cells accumulate several intermediary metabolites that may be shunted to interconnected pathways to support the biosynthesis of essential macromolecules and rapid proliferation (11, 30). One possible direction is the pentose phosphate pathway, while glyceraldehyde-3-phosphate, 3-phosphoglycerate, and fructose-6-phosphate are critical for the *de novo* synthesis of amino acids, phospholipids, and ribonucleotides, respectively (25, 31).

Increased glutamine demand and consumption are also important characteristics of the Warburg effect. Among transcription factors, MYC has been proven to induce glutamine transporters as well (25). Cancer cells utilize glutamine to fuel biosynthesis of nucleotides, to reload tricarboxylic acid cycle (TCA) intermediates, or to convert glutamine into lactate by the stepwise process of glutaminolysis (32). Besides biosynthesis, glutamine is also utilized for antioxidant defense function, since glutamine metabolism results in the concomitant production of NADPH, decreasing concentration of reactive oxygen species (ROS) and increasing glutathione levels, thereby protecting cells from oxidative stress (25, 33).

In malignantly transformed cells some tumor specific isoforms of metabolic enzymes also have been described. Opposite to normal cells that express pyruvate kinase M1 isoform (PKM1), tumor cells primarily express the M2 isoform that is responsible for the regulation of the final and rate-limiting reaction of the glycolytic pathway (34). PKM2 acts as a coactivator of HIF1α, inducing the expression of pyruvate dehydrogenase kinase (PDK) and lactate dehydrogenase (LDH) (34). Hexokinase II (HK II) is another example of tumor specific enzyme isoforms promoted by Akt protein (23).

Besides the tumor specific isoforms, the mutations of some metabolic enzymes also contribute to the metabolic switch featuring cancer cells. Gain of function mutations of isocitrate dehydrogenase (IDH) result in novel enzymatic activity and the production of the oncometabolite 2-hydroxyglutarate (2-HG) (35). Mutations of succinate dehydrogenase (SDH) and fumarate hydratase (FH) lead to the accumulation of succinate and fumarate, respectively, which inhibit α-ketoglutarate dependent enzymes including DNA-hydroxymethylating TET enzymes and Jumonji C domain containing histone demethylases (36). These mutations also contribute to the stabilization of HIF1α, due to the inhibition of α-ketoglutarate-dependent prolyl hydroxylase enzyme (37).

Cancer stem cells, the maintenance and self-renewal properties of which are tightly regulated by sirtuin enzymes, demonstrate unique metabolic flexibility. In general, they are characterized by even higher glycolytic rate (38), however, they can switch between glycolysis and oxidative phosphorylation in the presence of oxygen to maintain cellular homeostasis and promote tumor growth as well (39).

## Epigenetic Background of Warburg Effect With Emphasis on Sirtuin Enzymes

Development of Warburg-like metabolic phenotype of cancer cells is strongly regulated by epigenetic mechanisms where sirtuin enzymes play a key role. Various enzymatic activities, subcellular localizations and an enormous interactome with metabolic enzymes, epigenetic modifiers, and proteins of signal transduction pathways enable sirtuins to be central players in this field. In this section, we summarize data about the involvement of distinct sirtuins in the regulation of metabolism of cancer cells.

SIRT1 is a key metabolic sensor and regulator of mitochondrial biogenesis as well. SIRT1 and AMP-activated protein kinase (AMPK) directly activate the nuclear receptor Peroxisome Proliferator Activated Receptor Gamma Coactivator 1 alpha (PGC1α) through deacetylation and phosphorylation, respectively, resulting in increased mitochondrial biogenesis (40). In glioblastoma multiforme, activation of PGC1α leads to the differentiation of cells into a mature phenotype by activating some transcription factors related to mitochondrial biogenesis, therefore counteracting the Warburg effect (41).

In acute myeloid leukemia (AML) cell models, SIRT2 promoted the Warburg effect by deacetylating and activating glucose-6-phosphate dehydrogenase (G6PD) enzyme, increasing the production of NADPH, and supporting the biosynthesis of macromolecules that are essential for rapid cell proliferation (42). However, SIRT2 also deacetylates and destabilizes the ATP-citrate lyase (ACLY) enzyme (43), the low expression level of which is associated with favorable overall survival in AML patients (44). In cholangiocarcinoma, SIRT2 induces Warburg-like metabolic reprogramming resulting in decreased oxidative phosphorylation and increased activity of the serine synthesis pathway, consequently protecting cholangiocarcinoma cells from oxidative stress and apoptosis (45).

SIRT3 is the best characterized mitochondrial sirtuin enzyme, its expression level is increased by caloric restriction, fasting, and exercise training (9). The first reported target of SIRT3 was acetyl-CoA synthetase 2 (AceCS2) (46), that promotes metastasis formation of renal cell carcinoma (47), however, in gastric cancer, loss of AceCS2 expression predicts poor prognosis (48), which further emphasizes the significance of TME. SIRT3 down-regulates HIF1α and pyruvate dehydrogenase kinase 1 (PDK1) in cholangiocarcinoma (49), and suppresses the Warburg effect also by the activation of pyruvate dehydrogenase complex (PDC) and the indirect inhibition of the tumor specific isoenzyme hexokinase II (HK II) (11). SIRT3 regulates fatty acid oxidation via the deacetylation and activation of long chain acyl-CoA dehydrogenase (LCAD) enzyme (50), opposite to HIF1α that suppresses fatty acid oxidation to facilitate cancer progression (51). In cancer cells, similarly to the reduction of ATP production, the decrease of mitochondrial ROS formation is also significant, in which SIRT3 has been proved to play a pivotal role by the deacetylation and activation of superoxide dismutase 2 (SOD2) enzyme (52). Though SIRT3 promotes mitochondrial biogenesis (53), it also protects mitochondrial DNA from oxidative damage because of the deacetylation of 8-oxoguanine-DNA glycosylase 1 (OGG1), an enzyme that is involved in the process of DNA repair (54). Despite the fact that SIRT3 counteracts the Warburg effect in several ways, Warburg-promoting effect of this enzyme has also been confirmed. SIRT3 deacetylates and activates mitochondrial glutamate dehydrogenase (GDH) (55), therefore contributing to enhanced glutaminolysis, that is characteristic of Warburg effect. The rate-limiting enzyme of ketone body formation, 3-hydroxy-3-methylglutaryl-CoA synthase 2 (HMGCS2) is also regulated by SIRT3 (56). HMGCS2 is a tumor suppressor in prostate cancer, the knockdown of which promotes cell proliferation, colony formation, migration, and invasion of prostate cancer cells (57). SIRT3 was also found to deacetylate the serine hydroxymethyltransferase 2 (SHMT2) enzyme. Deacetylated SHMT2 is less stable, and indirectly counteracts the proliferation of colon cancer cells (58).

SIRT4 also has an impact on metabolism of cancer cells by promoting the Warburg effect. Besides the inhibition of glutamate dehydrogenase (GDH) enzyme (59), SIRT4 was described to inhibit malonyl-CoA decarboxylase, resulting in the repression of fatty acid oxidation (54). SIRT4 is also involved in the regulation of carbohydrate metabolism: according to novel findings, it increases the activity of PDC by repressing the expression of PDK1 enzyme (60). SIRT4 has also been described to regulate the leucine oxidation pathway (61). In a rat model, leucine-rich diet resulted in less glycolytic phenotype and decreased tumor aggressiveness (62).

SIRT5 regulates metabolic enzymes by deacetlyation, desuccinylation, deglutarylation, and demalonylation as well. Carbamoyl phosphate synthetase 1 (CPS1) is the only known protein that is deacetylated by SIRT5 (54). Elevated expression level of CPS1 would be supported by acetylation, and was associated with poor overall survival in LKB1-inactivated lung adenocarcinoma (63). SIRT5 desuccinylates and activates superoxide dismutase 1 (SOD1) (9), the oncogene serine hydroxymethyltransferase (SHMT2) (64), and pyruvate kinase M2 (PKM2) at two residues, K311 and K498 (8). Desuccinylation of PKM2 at K498 was shown to promote tumor development (8), while the role of K311 desuccinylation in cancer has not been described yet (65). In hepatocellular carcinoma, SIRT5 was shown to keep oxidative damage below toxic levels by desuccinylating and inhibiting peroxisomal acyl-CoA oxidase 1 (ACOX1) enzyme (66). In colorectal carcinoma cell lines, SIRT5 deglutarylates and activates glutamate dehydrogenase (GDH) enzyme, contributing to the Warburg effect (67). SIRT5 demalonylates and inactivates succinate dehydrogenase (SDH) enzyme leading to succinate accumulation, that results in the inhibition of α-ketoglutarate dependent dioxygenases as mentioned before (68).

SIRT6, which is one of the major epigenetic regulator of the glucose homeostasis of cells, exerts anti-Warburg effect by the inhibition of increased glucose uptake and overexpression of glycolytic enzymes as well. The latter results from the deacetylase activity of the enzyme, because H3K9 and H3K56 deacetylation of glycolytic genes inhibits their transcription by HIF1α and MYC, respectively (11). SIRT6 also inhibits hepatic gluconeogenesis by promoting the deacetylation of PGC1α transcription factor (69). Among tumor specific enzyme isoforms, SIRT6 deacetylates PKM2 at K433, leading to its nuclear export and the inhibition of PKM2 oncogenic functions (70).

SIRT7 has recently been described as an enzyme with ADP-ribosyl transferase activity (71). SIRT7 controls mitochondrial biogenesis, increases hepatic lipid accumulation and enhances adipogenesis in white adipocytes (72), however, its role in Warburg effect has yet to be examined.

Besides sirtuins, microRNAs are also important epigenetic regulators of the metabolic switch characteristic to cancer cells. MicroRNAs can either promote or inhibit the Warburg effect, depending on the target metabolic enzymes. MiR-26a promotes the Warburg effect by targeting pyruvate dehydrogenase X component (PDHX) in colorectal cancer cells, which inhibits the conversion of pyruvate to acetyl-CoA in the tricarboxylic acid cycle (TCA) (73). Both tumor suppressor and oncogenic microRNAs are involved in the regulation of tumor specific isoforms of some metabolic enzymes. Oncogenic miR-155 promotes the expression of hexokinase II, while tumor suppressor miR-124 inhibits the expression of pyruvate kinase M2 isoform (74). In non-small cell lung cancer cells, down-regulation of miR-214 inhibits glycolysis and proliferation, resulting from the decreased expression levels of hexokinase II and PKM2 enzymes (75). MiR-378^*^ contributes to the down-regulation of enzymes involved in TCA (22).

MicroRNAs and sirtuins cannot be regarded as independent regulators of the metabolism of cancer cells, since numerous interactions have been revealed between them. For example, miR-31 targets SIRT3 enzyme to increase oxidative stress in oral carcinoma (76).

## Anticancer Immunity and Its Connection With Altered Metabolic Phenotype: Role of Sirtuin Enzymes

It has been clear for more than a half century that one of the most important functions of the immune system is to identify and eliminate transformed cell clones (Macfarlane Burnet−1950) (77). On the other hand, tumors dampen antitumor immunity by several mechanisms, which is also a hallmark of cancer (25). Besides widespread crosstalk between immune cells and transformed clones, this hallmark involves a strong connection with metabolism as well: “if T cells play the music during an adaptive immune response, the metabolic tumor microenvironment calls the tune” (25, 78).

Cancer cells have been proved to induce decreased levels of nutrients and a hypoxic, acidic condition in the tumor microenvironment (25). Therefore, metabolic reprogramming is required by both tumor cells and immune cells in order to adapt to this microenvironment (78). However, adaptation results in the preference of different metabolic pathways in case of distinct cell types, in which sirtuin enzymes play an important role (Table 2).

**Table 2:** Characteristic metabolic phenotypes of cancer cells and immune cells of tissue microenvironment.

	**Cancer cell**	**Tumor-associated macrophage**	**Naive T cell**	**Activated T cell**	**Cytotoxic T lymphocyte**
Glycolysis	↑	↓		↑	↓
Oxidative phosphorylation	↓	↑	↑		↑
Tricarboxylic acid cycle	↓				
Fatty acid oxidation	↓	↑			
Glutaminolysis	↑			↑	
Production of ROS	↓				

T cells are the major components for antitumor immunity (78), however, a complex immunosuppressive network in cancer leads to the abrogation of immune and metabolic checkpoints, resulting in the limited activation and dysfunction of T cells (25). Similarly to cancer cells, T cells also undergo a metabolic switch upon their activation. Naive T cells rely mostly on oxidative phosphorylation and require relatively small amounts of glucose to maintain basic energetic demands (25). Though activated T cells engage in increased rates of glycolysis and glutaminolysis, it is important to note that opposite to transformed cells, this is the part of a physiological adaptation process in case of T cells (25). Metabolic intermediates generated by this metabolic switch are important for cytotoxicity and cytokine production as well (25). In recent years, sirtuins have been confirmed to regulate the differentiation and function of T cells in TME. SIRT1 negatively regulates the differentiation of IL-9-producing antitumor Th 9 cells in cancer (79) and suppresses the activity of regulatory T cells (80). On the contrary, SIRT3 has been proved to maintain immunosuppressive activity of regulatory T cells (80).

M2 polarized tumor-associated macrophages (TAMs) support tumor growth and provide a barrier against the natural killer (NK) cells and cytotoxic T lymphocytes (81). They exhibit up-regulated fatty acid synthesis and ß-oxidation (78), decreased glycolysis and the utilization of oxidative metabolism (82). TAMs are also characterized by high expression of IL-10, while they produce low levels of IL-12, tumor necrosis factor-α (TNFα) and inducible nitric oxide synthase (iNOS) (10). In hepatocellular carcinoma (HCC), SIRT1 was found to inhibit metastasis formation by promoting M1 macrophage polarization (83). Also in HCC, down-regulation of SIRT4 induces elevated monocyte chemoattractant protein-1 (MCP-1) expression, resulting in increased TAM infiltration of peritumor tissues (10).

Similarly to M2 polarized TAMs, cytotoxic T lymphocytes are also featured by a decreased rate of glycolysis and enhanced oxidative phosphorylation (78).

Central role of mitochondria in the maintenance of metabolic and redox homeostasis also has a strong impact on antitumor immunity. Mitochondrial oxidative metabolism was described as a critical suppressor of metastasis (84), while high levels of reactive oxygen species (ROS) in TME were confirmed to down-regulate the activity of antitumor effector T cells (85). Complexes of the electron transfer chain are regulated by the major mitochondrial deacetylase SIRT3, knockdown or deficiency of which correlates with decreased complex activity (86). In glioma stem cells, cooperative interplay between the mitochondrial chaperone TRAP1 and SIRT3 increases mitochondrial respiratory capacity and reduces the production of ROS (87).

Activation of the transcription factor aryl hydrocarbon receptor (AHR) induces the differentiation of CD4+ naive T cells into immunosuppressive regulatory T cells (88, 89). Besides playing critical roles in the initiation, promotion, progression, and metastasis of cancer (78), AHR is involved in the regulation of two NADases, contributing to decreased SIRT1 activity and the deregulation of glucose and fatty acid homeostasis as well (90). However, to our knowledge, interaction between AHR and sirtuin enzymes in cancer has not been established yet. There is also evidence that metabolites such as succinate and NAD^+^ are signals that regulate innate immunity, by acting via deacetylases such as SIRT1 and SIRT2 and regulating HIF1α, respectively (82).

## Special Aspects of Novel Epigenetic and Metabolic Therapeutic Approaches

In the twenty-first century, principles of personalized anticancer treatment need to involve the mapping of epigenetic and metabolic alterations, to both of which the contribution of sirtuin enzymes is fundamental. In parallel with this aim, growing number of antimetabolites and sirtuin inhibitors are administered. As an example for the latter, inhibition of SIRT1 by the up-regulation of miR-211-5p was associated with the induction of apoptosis in breast cancer cells (91). Inhibition of SIRT2 has been confirmed to induce the susceptibility of melanoma cell to the multikinase inhibitor dasatinib (92). Inactivation of SIRT3 leads to metabolic alterations, loss of stemness, and suppression of tumor formation by glioma stem cells *in vivo* (87).

Manipulating metabolism is also a tool to enhance antitumor immunity, since metabolic pathways have been proved to shape both function and survival of antitumor T cells (25). However, different metabolic interventions may be required in transformed clones and in distinct cell types of the immune system. For example, while inhibition of mevalonate metabolism in tumor cells attenuates the proliferation and growth of innate immune cells, the mevalonate pathway contributes to trained immunity as well (78).

It should also be considered, that inhibitors of sirtuin enzymes can induce different changes in distinct cell types of anticancer immunity. In myeloid cells, SIRT1 inhibition leads to increased transcription of proinflammatory cytokines, but on the other hand, targeting SIRT1 results in net immunosuppressive effect in case of T cells (93).

## Concluding Remarks and Future Perspectives

Sirtuins are key hubs in the regulatory network of etiological factors of tumors, having strong impact on the growth, survival, and metabolism of cancer cells, that also influences antitumor immunity in a significant manner. One of the major challenges of modern oncology is to establish novel elements of this regulatory-etiological network in order to provide personalized treatment for patients. Investigation of the exciting world of sirtuin enzymes is definitely one of the most effective tools for this. Further examinations are required to elucidate cell-specific metabolic and immunological effects of sirtuin inhibitors and activators. Appropriate vectors are also needed to deliver these small molecules and antimetabolites to their definitive target cells. Together with widespread genetic, epigenetic, and metabolic mapping at the time of the diagnosis, these efforts could improve therapeutic results, leading to longer disease-free and overall survival, with improved life quality as well.

## Author Contributions

Text of the manuscript was written by ZG with the supervision and instructions of LC.

### Conflict of Interest

The authors declare that the research was conducted in the absence of any commercial or financial relationships that could be construed as a potential conflict of interest.

## Abbreviations

2-HG: 2-hydroxyglutarate
AceCS2: Acetyl-CoA synthetase 2
ACOX1: acyl-CoA oxidase 1
AHR: aryl hydrocarbon receptor
AML: acute myeloid leukemia
AMPK: AMP activated protein kinase
CPS1: carbamoyl phosphate synthetase 1
EMT: epithelial-mesenchymal transition
FDG: fluorodeoxyglucose
FH: fumarate hidratase
FLT3: fms-like tyrosine kinase 3
G6PD: glucose-6-phoshate dehydrogenase
GDH: glutamate dehydrogenase
HAT: histone acetyltransferase
HCC: hepatocellular carcinoma
HDAC: histone deacetylase
HK: hexokinase
HMGCS2: 3-hydroxy-3-methylglutaryl CoA synthase 2
iNOS: inducible nitric oxide synthase
ITD: internal tandem duplication
LCAD: long chain acyl CoA dehydrogenase
LDH: lactate dehydrogenase
LKB1: liver kinase B1
LSC: leukemia stem cell
MCAD: medium chain acyl CoA dehydrogenase
MCP-1: monocyte chemoattractant protein-1
NSCLC: non-small cell lung cancer
NK: natural killer
OGG1: 8-oxoguanine-DNA glycosylase 1
PDC: pyruvate dehydrogenase complex
PDHX: pyruvate dehydrogenase X component
PDK: pyruvate dehydrogenase kinase
PET: positron emission tomography
PGM: phosphoglycerate mutase
PFK: phosphofructokinase
PGC1α: Peroxisome proliferator-activated receptor gamma coactivator 1-alpha
PKM2: M2 isoform of pyruvate kinase
RORγ: RAR-related orphan receptor gamma
ROS: reactive oxygen species
SDH: succinate dehydrogenase
SHMT: serine hydroxymethyltransferase
SIR2: Silent Information Regulator 2
SOD: superoxide dismutase
STAT3: signal transducer and activator of transcription 3
TAM: tumor associated macrophage
TCA: tricarboxylic acid cycle
TIGAR: tumor protein 53-induced glycolysis and apoptosis regulator
TME: tissue microenvironment
TNFα: tumor necrosis factor-α
TRAP1: tumor necrosis factor receptor associated protein 1.
